# Linking AKIN stages with tropical infection induced acute kidney injury among Indian patients

**DOI:** 10.6026/973206300220739

**Published:** 2026-02-28

**Authors:** Sharanya Kandaswamy, Moneshaa Srinivasan, Preethi Sekar, Jeevithan Shanmugam, Kaushik Gowthaman

**Affiliations:** 1 Department of Pathology, KMCH Institute of Health Sciences and Research, Coimbatore, India; 2 Department of General Medicine, Sri Venkateshwaraa Medical College Hospital and Research Centre, Ariyur, Pondicherry, India; 3 Department of Community Medicine, KMCH Institute of Health Sciences and Research, Coimbatore, India; 4 Consultant Neurologist, Kumaran Medical Center, Coimbatore, India

**Keywords:** Acute kidney injury (AKI), tropical febrile illness, leptospirosis, dengue, scrub typhus, AKIN criteria, renal failure, mortality predictors

## Abstract

Tropical acute febrile illness often causes AKI in India and outcome linkage with AKIN staging is still limited. Hence, we studied 150 adults with tropical infection related AKI staged by AKIN at 48 hours and followed till discharge or death. Leptospirosis was the main cause and higher AKIN stage showed more oliguria organ dysfunction longer stay higher dialysis need with mortality rising to 40.9% in stage III. This study adds a clear stage based risk gradient plus simple bedside red flags and basic labs for early triage and it supports timely aggressive support and dialysis referral to reduce avoidable deaths.

## Background:

Acute kidney injury is now seen as a major global problem. It increases in-hospital death and also future risk of chronic kidney disease and cardiovascular events [1]. Even in high income countries community-acquired AKI and hospital-acquired AKI show similar poor outcomes. This is seen when standard definitions are applied. So AKI burden is not only an ICU or surgical problem [2]. In tropical low and middle income regions, the pattern is different. Infections and acute febrile illnesses form a big share of AKI. This is not only sepsis or nephrotoxic drugs [3]. Monsoon epidemic PD-AKI data showed severe AKI was common with KDIGO stage 3 in 71% and dialysis in 29% of cases [4]. Studies on tropical acute febrile illness from Southeast Asia found leptospirosis, scrub typhus, dengue and malaria as leading causes. These infections are linked with longer stay and higher need of organ support [5]. Scrub typhus cohorts from India highlight that community-acquired AKI can be due to a single tropical infection. Delay in diagnosis increases risk of severe stage and death [6]. Recent Indian registry work on community-acquired AKI shows wide variation in etiology across centres. It stresses that more local data are needed for prevention and early referral strategies [7]. Rare case reports also remind that multiple concurrent tropical infections can occur in the same patient. They may precipitate severe multi-organ dysfunction including AKI [2]. Therefore, it is of interest to evaluate the clinical and biochemical profile of tropical infection induced AKI. We also aim to correlate AKIN stages with in-hospital outcomes in our setting.

## Materials and Methods:

This was a hospital-based descriptive study carried out in the Department of Internal Medicine, Sri Ramakrishna Hospital, Coimbatore. The study was conducted over a period of one year. Aim was to evaluate the clinical profile of acute kidney injury (AKI) in patients admitted with tropical acute febrile illness (TAFI) and to assess the prognostic value of Acute Kidney Injury Network (AKIN) staging.

## Study population:

All adult patients admitted with acute febrile illness of less than 21 days duration were screened. Tropical infections considered in this study included leptospirosis, dengue, malaria, scrub typhus and undifferentiated febrile illness, which are endemic in this region. Patients who fulfilled the diagnostic definition of AKI by AKIN criteria were included. Staging into AKIN I, II, III was done after 48 hours of admission based on serum creatinine and urine output. Patients requiring renal replacement therapy before 48 hours were directly classified as stage III.

## Inclusion and exclusion criteria:

Only patients giving written informed consent, or where consent could be obtained from the nearest kin, were enrolled. Patients with previous chronic kidney disease or renal pathology were excluded. Patients without consent were also excluded.

## Sample size was estimated using the formula for descriptive studies:

Sample size was calculated for a single proportion using n = (Z1-α/2)^2^ x P x (1-P) / d^2^, where Z1-α/2 = 1.96 for 95% confidence and d = 0.2P for 20% relative precision [8]. Minimum sample size came to 138. Finally here 150 patients were studied. For each patient detailed history and physical examination were done. Laboratory investigations included complete blood count, renal and liver function tests, serum electrolytes, ESR and disease-specific serologies. Patients were reviewed daily during hospital stay. Data regarding urine output, dialysis requirement and final outcome (recovery or death) were recorded. All data were entered into a pre-designed proforma. Quantitative variables were expressed as mean ± SD, categorical variables as proportions. Chi-square test was used for categorical comparisons and independent t-test for continuous variables. A p value ≤0.05 was taken as significant. Analysis was done with SPSS version 17.0. Approval was obtained from the Institutional Human Ethics Committee prior to data collection. Privacy and confidentiality were maintained throughout. Informed consent was taken from all patients or from relatives when the clinical condition prevented direct consent.

## Results and Discussion:

**Figure 1:**
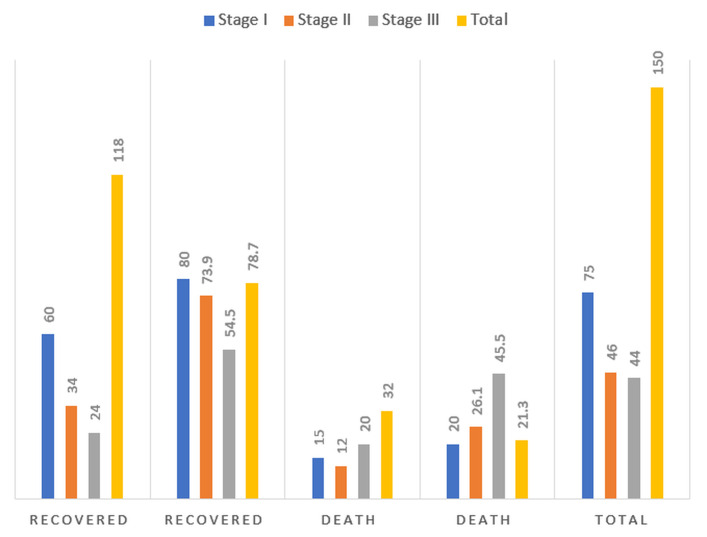
Distribution of acute kidney injury (AKI) stages according to AKIN criteria

**Figure 2:**
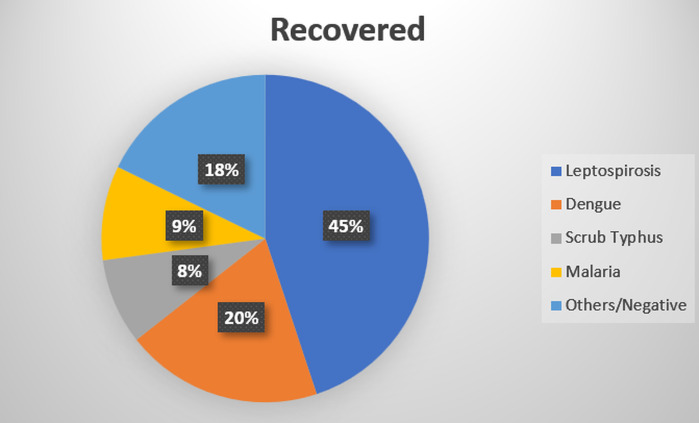
Etiological distribution among recovered AKI patients

Table 1 shows mean age 45.2 ± 15.1 years with male predominance 70.7% and rural majority 65.3% and low schooling up to 10th standard in 68.7% with alcohol use 34.6% and smoking 35.3%. This pattern is similar to South India tertiary care AKI cohort with mean age around 46 years and male predominance and largely community acquired rural profile [9] and it also matches Indian community acquired AKI cohorts where patients are younger and have less comorbidity than hospital acquired AKI [10]. Table 2 shows leptospirosis as the commonest cause 48% followed by dengue 16.7% undifferentiated AFI 16% malaria 10.7% and scrub typhus 8.6% with AKIN stage I 40% stage II 30.7% and stage III 29.3% as in Figure 1. Leptospirosis contributed a higher stage III share while dengue remained mostly stage I and a coastal Karnataka series reported dengue dominant tropical AKI with lower leptospirosis share showing regional seasonal variation [11]. Table 3 shows clinical severity clustering in stage III with oliguria bleeding tachypnoea shock and altered sensorium mainly in stage III and Table 4 show stepwise rise in WBC ESR bilirubin SGOT SGPT urea and creatinine with longer stay and higher dialysis sessions in stage III. This stage linked haemodynamic instability and multiorgan dysfunction pattern is consistent with ICU AKI severity class outcomes reported using KDIGO or RIFLE based staging [12, 13]. Table 5 shows clear stage wise outcome gradient with recovery falling from 96.7% in stage I to 73.9% in stage II and 59.1% in stage III and mortality reaching 40.9% in stage III. Oliguria, bleeding, tachypnoea shock and altered sensorium were strongly linked with death and this is comparable to RIFLE-based series where deaths clustered in the highest class and predictors included oliguria, haemodynamic failure, vasopressor and ventilation need which matches our high risk bedside signals in tropical AKI [14]. Figure 2 shows that leptospirosis was the predominant etiology among recovered AKI patients, accounting for 45% of cases. Table 6 shows non survivors had higher bilirubin, SGOT, SGPT urea and creatinine and required more dialysis sessions suggesting late presentation with advanced multi organ dysfunction and scrub typhus AKI is known to show jaundice with transaminitis where hepato renal involvement links with worse outcome and death risk [15]. Dengue AKI had lowest mortality 8% while leptospirosis malaria and scrub typhus had higher death rates and this aligns with reports that dengue AKI is often non oliguric needs dialysis less and recovers with early conservative care [16, 17]. This study clarifies that AKIN staging in tropical infection AKI predicts dialysis need length of stay and in hospital death with a steep rise in mortality in stage III. It also provides a practical triage set for routine wards using oliguria respiratory distress shock altered sensorium and basic labs like bilirubin urea, creatinine and transaminases. It adds etiology wise stage and mortality patterns for leptospirosis dengue, malaria and scrub typhus in one cohort which helps early referral and dialysis planning in rural community acquired cases. Despite single centre design AKIN use and no long term follow up the stage linked clinical and lab signals remain useful for early risk recognition in resource limited settings.

## Conclusion:

Tropical infections mainly leptospirosis were the leading cause of AKI in among young rural patients. Higher AKIN stages showed clear step up in dialysis need hospital stay and mortality with most deaths in stage III. Oliguria, bleeding, tachypnoea shock, altered sensorium and higher bilirubin, urea and creatinine reliably marked the highest risk group. Early recognition of these features with prompt supportive care and timely dialysis referral can still reduce avoidable deaths in tropical infection induced AKI.

## Figures and Tables

**Table 1:** Baseline demographic profile and general characteristics

Variable	n (%) / Mean±SD
Age (years, mean±SD)	45.2±15.1
Male sex	106 (70.7)
Female sex	44 (29.3)
Residence - Urban	52 (34.7)
Residence - Rural	98 (65.3)
Education ≤10th standard	103 (68.7)
Alcohol use	52 (34.6)
Smoking	53 (35.3)

**Table 2:** Etiological and AKIN stage distribution

Etiology	n (%)	AKIN Stage I; n (%)	AKIN Stage II; n (%)	AKIN Stage III; n (%)
Leptospirosis	72 (48.0)	18 (25.0)	23 (31.9)	31 (43.1)
Dengue fever	25 (16.7)	16 (64.0)	6 (24.0)	3 (12.0)
Malaria	16 (10.7)	7 (43.8)	6 (37.5)	3 (18.7)
Scrub typhus	13 (8.6)	6 (46.1)	5 (38.5)	2 (15.4)
Undifferentiated AFI	24 (16.0)	13 (54.2)	6 (25.0)	5 (20.8)
Total	150 (100)	60 (40.0)	46 (30.7)	44 (29.3)

**Table 3:** Clinical manifestations observed in different stages of AKIN

Clinical Feature	Stage I n (%)	Stage II n (%)	Stage III n (%)	p-value
Icterus	25 (26.9)	25 (26.9)	43 (46.2)	0.021
Oliguria	2 (3.4)	22 (37.3)	35 (59.3)	<0.001
Bleeding	6 (23.1)	5 (19.2)	15 (57.7)	0.018
Tachycardia	20 (21.5)	31 (33.3)	42 (45.2)	0.006
Tachypnoea	12 (20.0)	23 (38.3)	25 (41.7)	0.021
Shock	1 (5.0)	0 (0)	19 (95.0)	<0.001
Altered sensorium	1 (5.0)	0 (0)	19 (95.0)	<0.001

**Table 4:** Laboratory findings and biochemical parameters compared across AKIN stages

Parameter	Stage I Mean±SD	Stage II Mean±SD	Stage III Mean±SD	p-value
Hemoglobin (g/dL)	12.3±1.7	12.0±1.8	11.1±1.8	0.001
WBC (/mm^3^)	9509±3754	10622±3289	12836±4536	<0.001
Platelets (/mm^3^)	129216±57732	136055±50476	153904±49047	0.063
ESR (mm/hr)	36.3±25.1	43.8±23.5	55.8±25.1	<0.001
Serum Bilirubin (mg/dL)	2.38±2.03	3.35±2.8	6.70±4.1	<0.001
SGOT (U/L)	115.7±118.5	163.7±136.0	338.0±217.2	<0.001
SGPT (U/L)	126.6±130.6	174.6±135.7	351.4±220.3	<0.001
Blood Urea (mg/dL)	56.4±8.6	96.0±13.4	186.6±39.0	<0.001
Serum Creatinine (mg/dL)	1.71±0.19	3.43±0.55	6.27±1.82	<0.001
Hospital stay (days)	7.5±2.6	11.2±3.2	15.4±4.0	<0.001
Dialysis sessions	0	2.4±2.6	7.0±2.4	<0.001

**Table 5:** Outcome of illness in relation to AKIN stage and associated clinical predictors

Variable	Recovery n (%)	Death n (%)	p-value
AKIN Stage I	58 (96.7)	2 (3.3)	<0.001
AKIN Stage II	34 (73.9)	12 (26.1)	
AKIN Stage III	26 (59.1)	18 (40.9)	
Oliguria present	43 (72.9)	16 (27.1)	0.021
Bleeding	10 (38.5)	16 (61.5)	<0.001
Tachypnoea	36 (60.0)	24 (40.0)	<0.001
Shock	1 (5.0)	19 (95.0)	<0.001
Altered sensorium	2 (10.0)	18 (90.0)	<0.001

**Table 6:** Laboratory and etiological predictors of mortality

Parameter	Recovery Mean±SD	Death Mean±SD	p-value
Serum Bilirubin (mg/dL)	3.29±2.45	6.36±5.29	<0.001
SGOT (U/L)	170.4±154.4	288.8±245.4	0.001
SGPT (U/L)	177.6±147.6	316.8±268.7	<0.001
Blood Urea (mg/dL)	95.9±54.0	146.6±59.9	<0.001
Serum Creatinine (mg/dL)	3.08±1.75	5.39±2.49	0.001
Dialysis sessions	1.95±2.82	5.94±3.96	<0.001
Etiology	Recovery n (%)	Death n (%)	p-value
Dengue fever (n=25)	23 (92.0)	2 (8.0)	<0.001
Leptospirosis (n=72)	53 (73.6)	19 (26.4)	0.43
Malaria (n=16)	11 (68.8)	5 (31.2)	<0.001
Scrub typhus (n=13)	10 (76.9)	3 (23.1)	<0.001
